# Early antibody response against *Mycobacterium avium *subspecies *paratuberculosis *antigens in subclinical cattle

**DOI:** 10.1186/1477-5956-6-5

**Published:** 2008-01-28

**Authors:** John P Bannantine, Darrell O Bayles, W Ray Waters, Mitchell V Palmer, Judith R Stabel, Michael L Paustian

**Affiliations:** 1 Bacterial Diseases of Livestock Research Unit, USDA/ARS/National Animal Disease Center, Ames, IA 50010, USA

## Abstract

**Background:**

Our laboratories have previously reported on the experimental infection of cattle with *Mycobacterium avium *subsp *paratuberculosis *(*M. paratuberculosis*) using an intratonsillar infection model. In addition, we have recently developed a partial protein array representing 92 *M. paratuberculosis *coding sequences. These combined tools have enabled a unique look at the temporal analysis of *M. paratuberculosis *antigens within the native host. The primary objective of this study was to identify *M. paratuberculosis *antigens detected by cattle early during infection. A secondary objective was to evaluate the humoral immune response in cattle during the initial year of infection.

**Results:**

Sera from two experimentally infected cattle, taken pre-inoculation and at day 70, 194 and 321 post infection, identified dynamic antibody reactivity among antigens with some showing an increased response over time and others showing declining levels of reactivity over the same time period. A *M. paratuberculosis *specific protein, encoded by MAP0862, was strongly detected initially, but the antibody response became weaker with time. The most reactive protein was a putative surface antigen encoded by MAP1087. A second protein, MAP1204, implicated in virulence, was also strongly detected by day 70 in both cattle. Subsequent experiments showed that these two proteins were detected with sera from 5 of 9 naturally infected cattle in the subclinical stage of Johne's disease.

**Conclusion:**

Collectively these results demonstrate that *M. paratuberculosis *proteins are detected by sera from experimentally infected cattle as early as 70 days after exposure. These data further suggest at least two antigens may be useful in the early diagnosis of *M. paratuberculosis *infections. Finally, the construction and use of a protein array in this pilot study has led to a novel approach for discovery of *M. paratuberculosis *antigens.

## Background

Johne's disease is an economically significant intestinal disease caused by *Mycobacterium avium *subsp *paratuberculosis *(*M. paratuberculosis*). A recent survey estimated that 20%–40% of dairy herds in the United States are infected with *M. paratuberculosis *and producers lose $227 USD annually for each infected animal [1]. These costs are mostly attributed to the decreased milk production and weight loss resulting from the disease.

After *M. paratuberculosis *infection by ingestion of contaminated milk or grass containing fecal material from a shedding cow, there is a lengthy subclinical phase that can last for several years. During this stage the cows may appear healthy, but can intermittently shed low numbers of mycobacteria in the feces, enabling transmission to other animals including wildlife species. A major challenge in controlling Johne's disease is the ability to detect infected cattle prior to appearance of disease signs, such as diarrhea and heavy fecal shedding of *M. paratuberculosis*. An unknown trigger, possibly stress during lactation or parturition, advances the disease from subclinical to clinical where disease signs such as weight loss and diarrhea become evident [2,3]. This trigger appears to coincide with a shift in immune function from a Th1 response to a Th2 response [4]. Current detection of subclinical animals depends on the timing and sensitivity of the test. Even the most sensitive culture-based tests will not detect *M. paratuberculosis *if a subclinically infected animal is not shedding bacilli at the time the fecal or milk sample is collected. *M. paratuberculosis *antigen induced interferon (IFN)-γ has been shown to be elevated in subclinical animals, but this cytokine declines in the clinical stage concomitant with an increase in *M. paratuberculosis *specific IL-10 production [5,6]. A comprehensive cytokine profile has been reported for both circulating monocytes and local tissues obtained from *M. paratuberculosis*-infected cattle [7].

With a few notable exceptions [8-10], there is very little data on antibody detection of *M. paratuberculosis *at early stages of infection in cattle. There are several reasons for this, but one in particular is that cattle that appear healthy are not routinely evaluated using serial test bleeds and analysis. Furthermore, there are numerous studies that show the cell-mediated immune response in cattle predominates during the early stages of infection and is responsible for the initial control of this infection [4,6,11]. However, despite the lack of data describing the temporal detection of specific antigens by host antibodies early post infection, these experiments are critical to gain a better understanding of the pathogenesis, diagnostics and vaccine strategies for Johne's disease. For example, the ideal diagnostic antigen would be detected early and remain easily detected throughout the course of the disease. Alternatively, a good vaccine candidate antigen might only be detectable by antibody at a specific stage of the disease. Thus far, no such antigen has been discovered for Johne's disease.

The recent literature has revealed an emphasis on developing consensus animal models for Johne's disease study. One publication [12] is a result of an international meeting, sponsored by the Johne's Disease Integrated Program in the United States, which had the goal of proposing consensus animal models for each of the commodity groups including sheep and cattle as well as farmed deer in New Zealand. Detailed methods including dose, route, length of time for disease signs to appear, etc. were determined [12]. A second communication, published independently around the same time, provided similar information [13]. Prior to these comprehensive animal model reviews, we published a study describing the intratonsillar route of infection with *M. paratuberculosis *in neonatal calves [9]. Serial test bleeds were collected over the course of the 321-day study and analyzed for T cell and B cell responses using a variety of immunological assays. Selected test bleeds from that study were used on the protein array in the current study. The health status of these calves was further monitored using additional tests, including fecal culture and IS900 PCR. Although *M. paratuberculosis *was detected in all calves by 271 days post challenge, no clinical signs were observed in any animal throughout the study [9]. However, an antibody response was observed by 134 days post infection using a lipoarabinomannan-based ELISA test. A second study that evaluated an experimental infection of goats observed an antibody response as soon as 180 days post infection [14].

The study described herein combines the intratonsillar infection model [9] with newly developed protein array tools to obtain a temporal picture of antigen detection during the initial year of infection in cattle. While the concept of protein arrays has been around nearly as long as the DNA array, there are very few protein arrays available. This is because the process of constructing these arrays involves the production of dozens of specific proteins, a rate limiting and labor-intensive step when compared to oligonucleotide synthesis used in constructing DNA arrays. Nonetheless, studies using bacterial protein arrays are beginning to appear in the literature [15-17] due to the power provided by this tool for parallel protein analyses. Our laboratory initially developed a 48 spot protein array consisting of purified recombinant proteins representing selected *M. paratuberculosis *gene products [18]. That array was expanded to a 96-spot array and used in this study.

## Results

### Construction of a partial *M. paratuberculosis *protein array

A 96-spot protein array was constructed on nitrocellulose filters using a dot blot apparatus to imprint the array. Represented among these spots are 92 *M. paratuberculosis *recombinant fusion proteins including hypothetical proteins (20), known antigens (6), membrane proteins (10), and proteins with no similarity in public sequence databases (25). The affinity tag present within these recombinant fusion proteins is the maltose binding protein (MBP). MBP was also expressed as a fusion with the *E. coli *LacZ alpha peptide and used as a control to determine antibody reactivity to this tag in each experiment. In addition, there are two spots that contain a sonicated whole cell extract of *M. paratuberculosis *and one spot with only spotting buffer as a negative control. A complete listing of the proteins present on the array along with their sizes, concentration and predicted function are listed in Table 1.

**Table 1:** *Mycobacterium avium *subsp *paratuberculosis *proteins used in this study

Protein name^a^	Concentration (mg/ml)^b^	Gene size^c^	Amino acids^d^	Predicted function
MAP sonicate	1.0	NA^e^	NA	NA
MAP0075	0.18	423	140	conserved small membrane protein
MAP0087-his	1.0	717	238	probable secreted protein
MAP0105c	1.24	1058	352/889	hypothetical protein
MAP0182c	0.67	411	136	conserved hypothetical protein
MAP0216	1.66	1028	342/347	antigen 85A, mycolyltransferase
MAP0261c	0.49	465	154/161	19 kDa protein
MAP0389	2.04	947	315/337	diarylpropane peroxidase (EC 1.11.1.14) [Nostoc]
MAP0736	5.73	744	247	hypothetical protein
MAP0852	10.4	546	181	no BLAST hits
MAP0853	4.04	660	219	no BLAST hits
MAP0855	0.34	926	308/314	no BLAST hits
MAP0857c	0.70	318	105	hypothetical protein
MAP0858	0.44	537	178/182	no BLAST hits
MAP0859c	0.66	609	202	Mycobacterium phage L5
MAP0860c	0.04	885	294/296	no BLAST hits
MAP0861	0.43	342	113	no BLAST hits
MAP0862	0.48	989	329/360	no BLAST hits
MAP0863	0.66	675	224	no BLAST hits
MAP0864	7.42	426	141	no BLAST hits
MAP0865	0.08	1272	423	Hypothetical 30.9 kDa protein
MAP0866	3.07	804	267	integrase
MAP0904	0.81	371	124/246	conserved hypothetical protein
MAP0961c	0.18	719	239/352	hypothetical protein
MAP1087	0.43	441	146	prob. peptide transp. system permease, N-terminal
MAP1121c	0.32	498	165	putative substrate binding protein
MAP1174c	1.64	756	251	glucose-6-phosphate 1-dehydrogenase
MAP1204	2.61	735	244	putative exported p60 protein homologue
MAP1233	0.04	723	240	conserved hypothetical protein
MAP1345	0.69	600	199	no BLAST hits
MAP1388	0.08	396	131	no BLAST hits
MAP1416c	0.45	666	221	no BLAST hits
MAP1417c	0.54	435	144	no BLAST hits
MAP1609c	0.05	987	328/330	antigen 85B, mycolyltransferase
MAP1636c	1.12	425	141/157	no BLAST hits
MAP1643	0.45	2277	759/762	isocitrate lyase, [beta] module
MAP1655c	0.93	381	126	hypothetical protein
MAP1730c	0.26	1023	340	putative ATP/GTP-binding protein
MAP1931c	0.32	1104	367	anthranilate phosphoribosyltransferase
MAP2077c	1.66	330	109	hypothetical protein
MAP2116c	0.27	1269	422	cell invasion protein
MAP2121c	7.14	887	295/307	major membrane protein I
MAP2121c-his	0.99	924	307	major membrane protein I
MAP2151	2.79	438	145	no BLAST hits
MAP2155	9.57	312	103	similar to IS6110
MAP2156	0.59	963	320	putative transposase GEN: X52471)
MAP2157	0.38	1221	406	IS900
MAP2158	2.40	582	193	no BLAST hits
MAP2182c	0.26	435	144	conserved hypothetical protein
MAP2231	0.31	1422	473	PKS-associated protein, unknown function
MAP2360c	0.13	534	177	hypothetical protein
MAP2380	2.00	600	200/550	acyl-CoA synthase
MAP2657	0.26	651	216	putative oxidoreductase
MAP2663c	0.03	510	169	putative integral membrane protein
MAP2676c	1.30	354	117	hypothetical protein
MAP2734	0.37	1341	446	putative dioxygenasesdiooxygenases
MAP2740	0.24	666	221	putative membrane protein
MAP2751	0.70	582	193	no BLAST hits
MAP2753	0.17	309	103/252	hypothetical protein
MAP2761c	0.03	692	230/238	no BLAST hits
MAP2762c	1.45	425	141/146	no BLAST hits
MAP2764c	0.10	290	96/149	no BLAST hits
MAP2765c	0.06	539	179/396	no BLAST hits
MAP2767c	0.42	473	157/183	no BLAST hits
MAP2963c	0.88	1499	499/874	no BLAST hits
MAP3084c-his	1.0	684	227	probable secreted protein
MAP3121	0.38	855	284	enoyl-CoA hydratase/isomerase superfamily
MAP3129	0.09	404	134/141	hypothetical protein
MAP3131	0.30	1096	365/942	conserved large membrane protein
MAP3155c	1.22	273	90	hypothetical protein
MAP3434	0.18	690	230/330	possible membrane protein
MAP3437c	0.29	843	279/280	no BLAST hits
MAP3531c	0.28	1034	344/352	antigen 85C, mycolytransferase
MAP3734c	0.19	1559	519/593	putative ABC transporter ATP-binding protein
MAP3735c	0.42	1181	393/429	part of heavy metal tolerance protein
MAP3743	0.85	998	332/348	hypothetical protein
MAP3751	0.11	879	293/979	conserved large membrane protein
MAP3753	0.64	1353	450	hypothetical protein
MAP3761c	0.07	729	242	conserved membrane protein
MAP3771	0.02	294	97	50S ribosomal protein L31
MAP3817c	0.65	939	312	no BLAST hits
MAP3833c	0.13	626	208/260	hypothetical protein
MAP3840	0.10	1872	623	70 kD heat shock protein, chromosome replication
MAP3902c	0.15	522	173	Serine/threonine kinase (EC 2.7.1.37) [Myc...
MAP3903c	0.42	531	176	Serine/threonine kinase (EC 2.7.1.37) [Myc...
MAP3954	0.05	279	92	conserved hypothetical protein
MAP4014	0.21	951	316	probable 4-amino butyrate transporter, N-terminal
MAP4025	0.32	854	284/325	cytochrome c-type biogenesis protein
MAP4129	0.26	965	321/336	ABC transporter
MAP4198	0.13	1283	427/441	SecY subunit of preprotein translocase
MAP4207c	5.28	686	228/231	probable ATP-binding transport protein
MAP4199	0.03	540/546	179/181	probable adenylate kinase
MAP4228	0.05	222	73	initiation factor IF-1

^a^Protein name corresponds to the genome project designation (Li et al., 2005). A second designation is included if known.

^b^Protein concentration is reported in as measured by Nanodrop spectrometry at 280_nm_.

^c^Gene size is reported in base pairs (bp). In cases where only a portion of the *M. avium *subsp *paratuberculosis *gene was cloned, the number of bp present in the clone is reported first followed by the number of bp in the full-length *M. avium *subsp *paratuberculosis *gene.

^d^Number of *M. avium *subsp *paratuberculosis *amino acids present in the fusion protein. In cases where only a portion of the *M. avium *subsp *paratuberculosis *protein is represented, the number of amino acids present is listed first followed by the total number of *M. avium *subsp *paratuberculosis *amino acids in the full-length protein.

^e^NA is not applicable.

The performance of the array was tested using a monoclonal antibody developed to the MBP tag as shown previously [18]. These experiments showed that all proteins containing the tag were readily detectable by immunoblot analysis (data not shown).

### Humoral immune response of experimentally infected cattle

**Figure 1:**
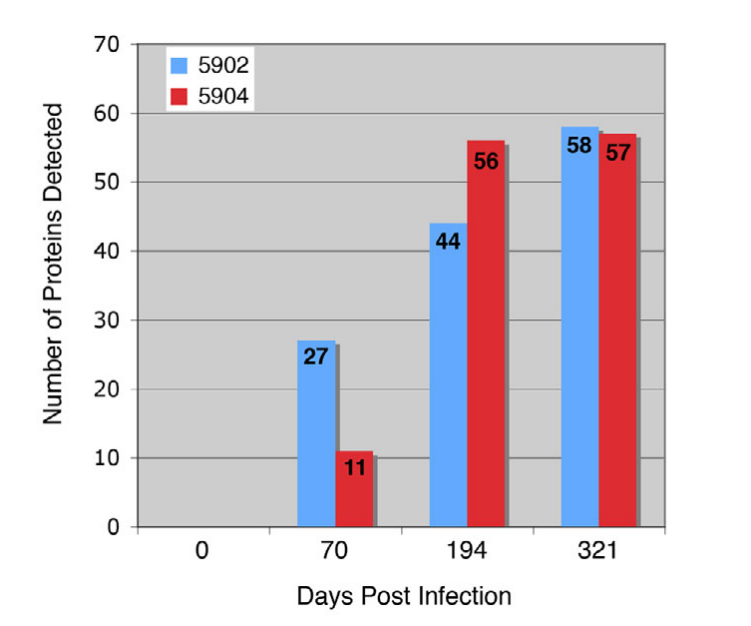
**Total number of *M. paratuberculosis *recombinant proteins detected by sera from experimentally infected cattle**. The graph shows the number of proteins on the 96-dot array (y-axis) that were detected at the day post infection (x-axis) for each calf. The total number of proteins detected is indicated on each bar. Note that similar numbers of proteins were detected by each animal at the end of the study period.

**Figure 2:**
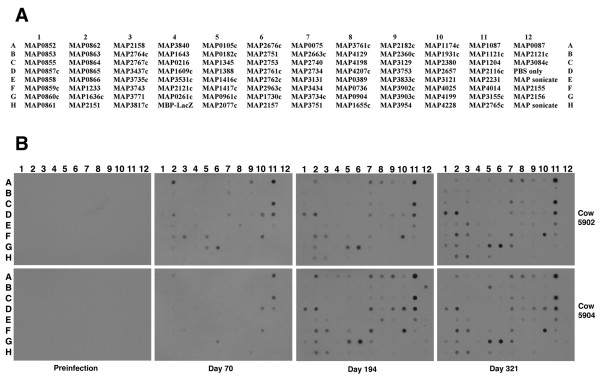
**Use of the dot blot protein array to obtain antibody reactivity profiles of experimentally infected calves**. Shown are the spot assignments for the array (A) and dot blot arrays exposed to sera from experimentally infected calves (B). The time point for when each serum sample was collected is indicated in the margin beneath the images. The animal number is listed in the right margin. A whole cell lysate representing a majority of the proteins produced by *M. paratuberculosis *is spotted in E12 and H12 for all dot blots. Three proteins present on the upper right corner of the array (in column 12) are polyhistidine tagged proteins (MAP0087, MAP2121c and MAP3084c). The remaining 89 spots contain MBP fusion proteins of *M. paratuberculosis *coding sequences. Note that the MAP2121c coding sequence is represented twice on the array; once as an MBP fusion (spot F4) and also a polyhistidine tagged protein (12B).

An analysis of serial bleeds collected from two experimentally infected calves was performed to temporally examine the humoral immune response to the arrayed *M. paratuberculosis *proteins. In general, the antibody response against the set of 92 proteins was remarkably similar between the two animals. One notable difference was that fewer proteins were recognized at day 70 in animal 5904 compared to 5902, as only 11 proteins compared to 27 proteins were detected, respectively (Figure 1 and Figure 2). However by day 194, animal 5904 had antibodies against more *M. paratuberculosis *proteins than did animal 5902. Serum from both animals detected similar numbers of proteins at the end of the study period (Figure 1). The pre-infection sera showed no detection of any of the recombinant proteins (Figure 2) and there was no detection of the MBP-LacZ protein for all time points suggesting that these calves do not have antibodies to the maltose binding protein (MBP) affinity purification tag.

### Temporal analysis of *M. paratuberculosis *proteins

**Figure 3:**
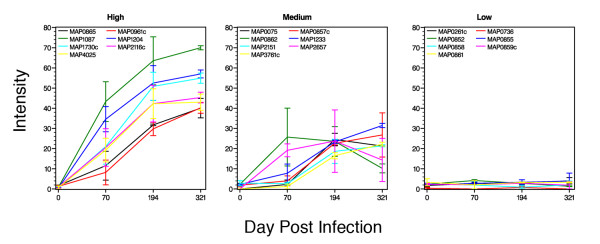
**Temporal trends of protein reactivity in experimentally infected cattle**. The lines show the average intensity for each protein over the course of the study period. The range observed between the two calves is indicated by statistical bars. Kernel Density Estimation performed on the log-transformed maximum intensity scores indicated there were three distinct normally distributed peaks. These distributions are statistically significant and define the proteins belonging to the weak, moderate, or strong response groups. The graph labeled high contains the seven proteins most strongly detected by the cattle sera and the graph labeled low contains the seven proteins that showed the least reactivity with cattle sera. The seven proteins in the medium category are also representative of reactive antigens that either showed low reactivity initially or declining reactivity with time.

The arrays exposed to cattle sera were analyzed by densitometry to obtain a quantitative measure of antibody reactivity (Table 2). When evaluating selected proteins over the course of the study, certain trends in reactivity appear to emerge for some proteins. For example, in both calves, antibody reactivity declined between day 194 and day 321 for a group of 11 proteins. This was especially true for MAP0862 and MAP2657, which showed the most severe decline among these 11 proteins (Table 2). The seven proteins that best fit into strong, moderate and weak response categories are shown in Figure 3. While strong and moderately detected proteins showed a wide range between the calves at days 70 and 194, this range was considerably reduced in the high category proteins at day 321 (Figure 3).

**Table 2:** Spot intensities of proteins present on the array.^a^

		Cow 5902				Cow 5904			
		
Spot Loc^b^	Protein	Day 0	Day 70	Day 194	Day 321	Day 0	Day 70	Day 194	Day 321
A7	MAP0075	0	4.472	21.431	21.122	0	0	27.612	21.36
A12	MAP0087-his	0.978	0	0	0	1.112	1.882	0	1.228
A5	MAP0105c	0	6.929	3.867	5.096	1.186	2.321	10.106	1.813
B5	MAP0182c	0	5.407	0	1.924	1.177	2.409	1.617	22.366
C4	MAP0216	0.652	3.779	1.296	1.395	2.175	3.444	0	0
G4	MAP0261c	2.395	2.287	3.487	5.668	1.097	3.146	2.397	2.496
E8	MAP0389	0	7.809	3.163	1.817	0	1.533	16.751	5.529
F8	MAP0736	0.734	0	0	0	0	0	1.446	0
A1	MAP0852	2.257	3.747	2.908	1.147	2.564	4.661	2.709	1.537
B1	MAP0853	2.144	3.734	5.095	8.497	1.961	4.158	3.239	2.649
C1	MAP0855	2.538	3.387	4.552	7.898	1.716	2.248	2.132	0
D1	MAP0857c	2.709	6.445	21.867	37.718	1.045	1.602	23.425	15.825
E1	MAP0858	3.005	3.882	1.775	3.999	1.978	0	0	0
F1	MAP0859c	2.821	4.137	2.87	3.745	1.909	0	2.803	0
G1	MAP0860c	2.996	0	5.531	12.743	1.592	0	7.669	6.676
H1	MAP0861	**5.074**^c^	0	3.407	3.053	1.546	4.05	3.111	3.52
A2	MAP0862	1.795	40.036	16.196	7.997	2.796	11.451	30.918	12.422
B2	MAP0863	1.518	5.524	3.174	4.592	2.057	4.891	3.274	1.473
C2	MAP0864	1.255	3.44	1.781	1.726	2.016	3.56	0	0
D2	MAP0865	1.909	18.708	32.138	44.846	1.317	4.386	31.361	35.251
E2	MAP0866	2.346	7.051	2.67	7.116	1.315	3.561	4.052	6.009
G8	MAP0904	1.678	0	0	0	0	0	0	0
G5	MAP0961c	1.797	14.366	26.5	37.546	0.78	2.103	33.046	42.877
A11	MAP1087	0.753	53.179	**51.634**	**70.968**	0.874	33.469	**75.392**	**68.964**
B11	MAP1121c	0.758	2.438	0	2.094	0.699	3.364	0	1.848
A10	MAP1174c	0	2.301	3.155	4.411	0.623	1.921	5.81	4.955
C11	MAP1204	0.78	40.8	43.856	55.046	0	28.367	61.122	58.985
F2	MAP1233	2.568	12.375	22.507	32.493	1.932	3.212	24.157	30.447
C5	MAP1345	0	3.339	1.861	1.018	1.147	0	3.413	2.389
D5	MAP1388	0	2.281	0	0	0.429	2.835	0	0
E5	MAP1416c	0	1.647	0	0	0.791	3.791	0	0
F5	MAP1417c	1.052	14.882	4.095	6.212	0.955	3.979	7.614	3.521
D4	MAP1609c	0.733	3.916	0	0	1.003	5.292	0	0
G2	MAP1636c	3.286	6.944	13.249	17.272	1.701	0	17.226	12.06
B4	MAP1643	0	4.155	0	1.534	1.827	0	0	0
H8	MAP1655c	2.788	0	0	0	0	2.049	0	0
G6	MAP1730c	1.147	29.831	43.872	52.35	0	12.272	57.799	57.47
B10	MAP1931c	0	2.917	0	2.217	0.511	2.886	2.077	3.339
H5	MAP2077c	3.833	0	2.784	9.443	1.385	0	3.63	9.221
D11	MAP2116c	1.418	29.88	33.215	42.633	0.568	11.24	51.336	47.918
F4	MAP2121c	1.76	4.158	1.373	1.513	0.969	3.005	0	0
B12	MAP2121c-his	1.247	0	0	0	0.889	2.172	21.226	2.155
H2	MAP2151	4.136	1.919	12.688	21.897	1.571	3.402	24.619	20.889
F12	MAP2155	2.434	0	1.385	0	1.251	2.039	0	2.105
G12	MAP2156	3.731	2.327	0	2.12	1.357	2.647	5.42	5.113
H6	MAP2157	3.504	0	0	0	0.751	1.532	1.51	2.683
A3	MAP2158	1.403	3.487	4.2	3.396	**3.187**	1.704	6.682	2.204
A9	MAP2182c	0	19.101	7.349	8.933	0	0	27.935	12.096
E11	MAP2231	1.846	3.732	11.54	15.671	1.166	0	17.203	18.933
B9	MAP2360c	0	3.168	0	0	0	2.766	0	0
C10	MAP2380	0.772	2.139	1.198	0	0	0	1.968	3.225
D10	MAP2657	0	22.372	8.29	3.68	0	15.937	39.178	25.032
B7	MAP2663c	0	9.594	3.661	2.248	0.868	0	4.9	2.717
A6	MAP2676c	0	3.291	1.406	0	0	0	2.036	0
D7	MAP2734	2.726	9.109	11.031	9.269	0	0	27.935	18.79
C7	MAP2740	0	1.736	6.447	18.061	0.507	0	6.295	10.804
B6	MAP2751	0	3.157	1.101	2.373	0.8	0	1.729	2.206
C6	MAP2753	0	1.909	0	2.176	0.624	0	1.701	2.585
D6	MAP2761c	0	0	0	0	0	0	0	0
E6	MAP2762c	0	0	0	0	0	2.397	0	0
B3	MAP2764c	1.472	2.739	1.969	5.874	2.13	5.442	2.039	1.955
H11	MAP2765c	4.816	0	0	0	0	4.799	0	0
C3	MAP2767c	1.257	3.507	2.303	7.019	1.982	4.347	1.92	2.722
F6	MAP2963c	0	0	0	0	0	2.204	0	0
C12	MAP3084c-his	1.313	0	0	0	0.999	2.026	0	1.982
E10	MAP3121	1.079	0	0	0	0	0	0	0
C9	MAP3129	1.406	2.366	0	1.206	0	0	1.731	2.865
E7	MAP3131	0	0	0	0	0	0	1.404	1.713
G11	MAP3155c	2.783	2.553	2.725	3.841	1.435	2.237	2.226	4.865
F7	MAP3434	0.832	1.401	8.312	19.522	0.508	0	33.289	14.418
D3	MAP3437c	1.674	3.321	1.29	1.587	1.359	1.965	0	0
E4	MAP3531c	0.926	7.373	2.472	4.313	0.558	2.834	1.374	0
G7	MAP3734c	1.085	2.238	2.719	17.809	0	0	10.914	20.81
E3	MAP3735c	1.944	4.897	5.078	18.618	1.326	3.368	3.887	4.502
F3	MAP3743	2.113	23.874	16.282	16.773	1.28	6.953	21.309	22.067
H7	MAP3751	2.991	0	0	0	0	0	2.499	3.033
D9	MAP3753	0	2.912	7.043	3.212	0	0	18.95	15.247
A8	MAP3761c	0	2.473	14.931	23.178	0	0	18.02	21.264
G3	MAP3771	2.81	2.229	1.344	2.528	0.953	1.997	0	0
H3	MAP3817c	3.836	2.526	10.298	22.826	1.101	1.552	15.736	19.901
E9	MAP3833c	1.078	0	2.07	2.755	0	0	3.676	4.636
A4	MAP3840	0.823	4.968	3.842	4.521	1.403	0	10.083	0
F9	MAP3902c	1.095	0	0	0	0	2.376	0	0
G9	MAP3903c	1.755	1.355	0	1.027	0	1.772	0	0
H9	MAP3954	3.426	0	0	1.969	0	7.584	1.612	3.629
F11	MAP4014	2.266	2.035	5.415	8.98	0.545	0	5.293	8.043
F10	MAP4025	1.474	25.139	34.876	39.022	0	13.413	49.655	47.012
B8	MAP4129	0	1.489	0	1.363	0	0	1.435	2.585
C8	MAP4198	0	1.536	1.517	6.817	0	0	5.431	6.175
G10	MAP4199	1.919	1.688	0	0	0.821	2.339	0	0
D8	MAP4207c	1.981	1.691	1.872	1.364	0	0	3.699	5.632
H10	MAP4228	4.373	0	0	0	0	3.122	0	0
H4	MBP-LacZ	4.128	0	0	1.722	1.303	0	0	0
D12	PBS buffer^d^	1.676	0	1.236	0	0.857	2.322	0	2.003
E12	Sonicate^e^	2.673	0	0	0	1.287	1.936	2.963	3.523
H12	Sonicate	5.01	1.359	0	0	1.366	4.337	3.002	4.472

^a^Listed are the intensity values for each spot arranged by ascending MAP number. Measurements were made using a consistent 1804 pixel area circle to record spot density using the Adobe Photoshop CS3 Extended application.

^b^Loc is location on the dot blot array.

^c^The strongest intensity for each time point is shown in bold.

^d^PBS buffer in spot D12 represents the spotting buffer only control.

^e^Sonicate is a sonicated whole cell lysate of *M. avium *subsp *paratuberculosis*.

### Identification of antigens detected early in *M. paratuberculosis *infection

Overall, the strongest antigen among this set of fusion proteins was encoded by MAP1087 (Figures 2 and 3). Antibody reactivity to this protein was observed in both animals by day 70 and remained the strongest detected protein throughout the course of the study (Figure 3 and Table 2). MAP1204, a putative p60 homolog, was also strongly detected by serum from both animals. Other antigens identified in these studies include MAP1730c, a putative ATP/GTP binding protein, and *M. paratuberculosis *hypothetical sequences MAP0865 and MAP0961c. MAP0865 contains the F57 DNA fragment which has previously been shown to be present uniquely in *M. paratuberculosis *[19] and resides on a 15.3 kb genomic island termed large sequence polymorphism 4 (LSP4) [20], making this protein an excellent candidate for use in a diagnostic test. Likewise, MAP0961c shows no sequence similarity in nucleotide databases and is present on a 13.5 kb segment of the genome termed LSP5 [20]. However, a similarity search of the SwissProt protein database reveals a glycosyltransferase domain that may be involved in cell envelope biogenesis. Furthermore, a corresponding ortholog, termed MUL_4677, was found in the recently published genome sequence of *Mycobacterium ulcerans *[21]. Therefore, caution must be used if this protein were to be considered for use in a diagnostic test. It is noteworthy that the whole cell sonicate antigen, which was spotted in duplicate on the protein array (12E and 12H), was not detected by either experimentally infected animal. This same antigen preparation reacted very strongly to anti-*M. paratuberculosis *sera from mice and rabbits previously [18]. In addition, two of three clinical cows showed an antibody response to the whole cell sonicate antigen [18] and hence it should be considered a potent preparation. This antigen preparation is likely similar to that used in the ELISA test.

### Early antigens are detected by sera from naturally infected cattle in the subclinical stage of Johne's disease

**Figure 4:**
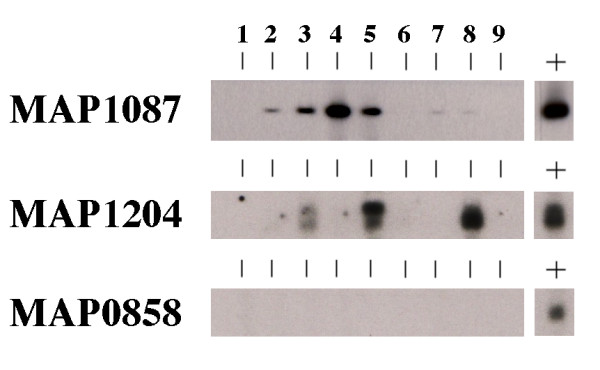
**Slot immunoblot analysis of selected recombinant proteins with serum from cattle in the subclinical stage of infection**. A preparative, single well gel was loaded with the protein indicated in the left margin and blotted onto nitrocellulose filters. The filters were placed into a slot blotting device and 1:500 dilutions of each cattle sera were loaded into independent slots. Each blot was further processed as described previously [29]. The positive control, indicated by the lane with a "+" sign above it, was probed with a monoclonal antibody developed against the MBP affinity tag as described previously [29]. These results demonstrate that MAP1087 and MAP1204 are detected by sera from naturally infected cattle in the subclinical phase of infection. Serum sample slot assignments: 1, cow 85; 2, cow 112; 3, cow 113; 4, cow 117; 5, cow 183; 6, cow 235; 7, cow 834; 8, cow 871 and 9, cow 2715.

A panel of sera collected from 9 cows in the subclinical stage of infection was analyzed by immunoblot against the two strongest antigens identified from the protein array experiments. These animals are housed at the National Animal Disease Center and are monitored four times a year for shedding of bacilli and immunological status as measured by ELISA assays for antibody and IFN-γ (Table 3). MAP1087 was detected in 4 of 9 serum samples (Figure 4, lanes 2–5) and very weakly reactive with another 2 serum samples (Figure 4, lanes 7 and 8). Three of the nine serum samples from the naturally infected cows detected MAP1204. Serum from cow 183 detected both MAP1087 and MAP1204 proteins. Likewise, serum from cow 113 detected both proteins although weakly for MAP1204. Neither antigen was detected by sera from three of the cows tested (Figure 4, lanes 1, 6, and 9). Finally, none of the sera from these animals detected the protein encoded by MAP0858, a protein that was also not detected by the experimentally infected cattle sera (Figure 2). These data suggest that MAP1087 and MAP1204 proteins may be detectable in *M. paratuberculosis*-infected animals prior to the clinical stage of disease. In conclusion, we hypothesize that while these antigens, if combined, would recognized by 66% of the subclinical cows, additional antigen(s) need to be included along with MAP1204 and MAP1087 in order to detect all nine subclinical cows.

**Table 3:** Culture and immunological status of cattle used in this study.

			IFN-γ (mg/ml)^c^			
				
Cow ID	Survey date^a^	Fecal culture (CFU)^b^	NS	ConA	MPS	ELISA (S/P ratio)^d^
85	12/20/05	C, C, C, C	0.069	0.454	0.278	0.002
	3/4/06	2, 2, 2	0.061	0.185	0.068	ND
112	12/20/05	C, C, 21, 1	0.07	0.218	0.064	0.027
	4/10/06	2, 1, 1	0.063	1.83	0.09	0.014
113	8/10/04	C, C, C, C	0.21	0.514	0.054	0.041
	11/10/04	0, 3, 1	0.39	1.51	0.54	0.09
117	12/17/04	T, T, T, T	0.053	0.143	0.097	-0.052
	3/1/05	T, C, C	0.039	0.174	0.046	ND
183	12/20/05	0, 0, C, C	0.054	0.241	0.145	1.42
	4/7/06	3, 2, 0	0.089	1.134	0.194	0.981
235	12/20/05	0, 0, 0, 0	0.068	0.362	0.114	0.039
	3/4/06	0, 1, 1	0.05	0.35	0.064	0.021
834	8/10/04	0, 1, 0, 1	0.033	0.336	0.142	0.87
	11/11/04	0, 0, 0	0.044	0.806	0.076	0.648
871	8/10/04	C, C, C, C	0.037	0.361	0.054	0.049
	12/21/04	C, 2, 1	0.043	0.189	0.064	0.121
2715	12/20/05	2, 0, 1, 0	0.067	1.3	0.324	0.226
	3/18/06	0, 0, 0	0.059	0.211	0.062	0.044

^a^Each animal is surveyed quarterly to monitor progression of Johne's disease. The two most recent quarterly surveys are shown for each animal, but only sera collected on the most recent date was used for the immunoblot assay in Figure 4.

^b^Fecal culture is reported as numbers of colony forming units (CFU) present on each HYEM slant (platings are in triplicate or quadruplicate). A "C" indicates the slant had bacterial growth other than *M. paratuberculosis*. A "T" designation means too many colonies to count on that slant.

^c^The gamma interferon (IFN-γ) assay. Subclinical cows typically have a *M. paratuberculosis *sonicate (MPS) reading higher than the no stimulation (NS) reading, along with little or no colonies on fecal culture slants. The concanavalin A (ConA) reading is a positive control as cells are readily stimulated by this lectin protein.

^d^The ELISA sample to positive (S/P) ratio is calculated from the optical density reading as described previously [11].

## Discussion

Diagnosis of Johne's disease is difficult because as many as 90% of infected animals may show no signs of disease during sample collection and there is a lack of sensitive and specific assays that detect *M. paratuberculosis *early during infection. Antigen-based diagnostic tests for Johne's disease currently use a complex mixture of antigens, such as PPD or mycobacterial membrane fractions, that can exhibit cross reactivity with other mycobacteria [22]. Results from this study further suggest that using complex protein mixtures such as a whole cell sonicate extract may not be as sensitive when compared to a few recombinant proteins. This may be due to the heterogeneity of the sonicated extract where each protein is present in very small quantities. New scientific breakthroughs are expected from the recently completed genome sequence of *M. paratuberculosis *as this information now enables researchers to select and characterize *M. paratuberculosis *sequences of interest. Our interest is in characterizing those sequences that are novel and specific to *M. paratuberculosis *in order to develop the best available diagnostic tools. This study represents the most comprehensive *M. paratuberculosis *antigen analysis on a temporal scale. Data from this study provide a first look at how specific antibody reactivity changes over time and in direct comparison to other proteins.

When a temporal analysis of serial bleeds from experimentally infected calves was undertaken, we discovered a 50-kDa protein that was detected by day 14 post-challenge and a 60-kDa protein was evident by day 42 [9]. However, these studies were performed with a whole cell extract as the antigen and thus the identity of these early antigens were never discovered. Nonetheless, these studies showed that a humoral immune response was initiated in calves within a short time period following exposure to *M. paratuberculosis*. The current study extends these initial findings by using a recently developed protein array to probe sera from these experimentally infected calves. The two proteins that elicited the strongest antibody reactivity were MAP1087, a probable peptide transport system permease and MAP1204, a p60 protein homolog. These proteins have a calculated size of 15.4 kDa and 25.4 kDa, respectively. Therefore, it is unlikely that the proteins encoded by MAP1087 and MAP1204 are the same 2 antigens detected in the initial study by Waters et al [9]. Although MAP1204 is much more conserved across the mycobacterial genus, both proteins are present as orthologs in at least the genome sequences of *M. smegmatis *and *M. avium *strain 104. This raises the potential for cross-reactivity if these antigens were incorporated into a diagnostic test for Johne's disease.

A *M. paratuberculosis *protein array was initially constructed from 43 recombinant proteins and tested with sera from a variety of host species [18]. The protein array has since been expanded to include a total of 92 recombinant proteins for the current study. Additional experiments are planned that will use the protein array to examine antibody profiles of subclinical cattle versus clinical cattle. This study focused specifically on antigens detected early following infection of cattle with *M. paratuberculosis*. Performing a test to detect *M. paratuberculosis *antigens when clinical signs of the disease are already evident in the host, make the control of this disease by test and cull strategies futile. An optimized serological test that incorporates antigens detected at early times post-infection is a critical tool that animal producers/farmers need to control Johne's disease. In this way infected animals can be identified prior to shedding and exposure of herd mates to the disease causing bacterium. Regarding uninfected cows, one key advantage of the experimental model used is that each cow had its own animal-specific negative control (the preinfection bleed). This type of control is not available in most Johne's disease studies. In summary, a highly specific test that could identify infected animals within herds may have a greater and more immediate impact on cattle industries than any other current management approach, including vaccination.

The ideal *M. paratuberculosis *protein antigen likely remains undiscovered simply because all the proteins produced by *M. paratuberculosis *are not represented on the current protein array. In this study, promising candidates have been found, but the genome sequence annotation suggests that *M. paratuberculosis *produces a total of 4,530 proteins [23]. Therefore, until a larger percentage of the genome has been evaluated in a parallel manner similar to this study, better antigens are still likely to be discovered. Another limitation to using a protein array for antigen discovery involves the identification of epitopes that may be formed by multiple protein complexes. This possibility cannot be revealed when proteins are produced and spotted in isolation such as on an array.

Our laboratory, in collaboration with others, has successfully completed the genome sequence of this significant veterinary pathogen [23] and have used these data to identify coding sequences specific to *M. paratuberculosis*. Those studies have revealed less than 40 coding sequences that are uniquely present in *M. paratuberculosis *[24,25]. This surprisingly small number of unique coding sequences is primarily due to the genetic similarity among members of the mycobacteria [26]. Among these coding sequences, one gene, designated ISMAP02, is present in six copies distributed randomly in the genome [25,27]. Furthermore, we have evaluated the expression products from several of these *M. paratuberculosis*-specific sequences for immunoreactivity with sera from animals exposed to *M. paratuberculosis *[25,28]. Those studies identified proteins encoded by MAP0862, MAP2963c and MAP3732c as potential diagnostic antigens. The newly constructed *M. paratuberculosis *protein array has many of these same coding sequences represented. Both MAP0862 and MAP2963c were detected in each animal in this study, but MAP3732c is not present on the array used in the current study. The antigens identified in this study should be further evaluated with naturally infected animals in field trials before incorporation into a diagnostic test for Johne's disease.

## Conclusion

Of the 92 recombinant *M. paratuberculosis *proteins analyzed in this study, 2 emerged as potential antigens for the early detection of Johne's disease in cattle. These proteins are encoded by MAP1087 and MAP1204. The combination of MAP1087 and MAP1204 detected more subclinical cows than either protein alone, suggesting that the ideal detection antigen will comprise a mixture of more than one protein. Finally, this is a pilot scale study that demonstrates the method is effective for use in a large-scale antigen discovery project.

## Methods

### Animals

All cattle used in this study were housed at the National Animal Disease Center (NADC) and handled using institutional care guidelines set by the animal care and use committee. Experimentally infected calves were put in biosafety level 2 containment immediately following birth to prevent exposure to environmental mycobacteria. Naturally infected subclinical cattle are contained on a field pasture-barn set up on site at NADC. The experimentally infected animals consisted of male Holstein calves less than 1 year of age [9]. The health status and infection procedure have been reported previously [9]. Briefly, calves were infected with *M. paratuberculosis *by instillation of four weekly doses of approximately 4 × 10^6 ^CFU (in 0.2 ml PBS) into both tonsillar crypts weekly from 2 to 5 weeks of age. The subclinical cows used in this study comprised Holsteins, Brown Swiss and Guernsey with ages that ranged from 2–7 years.

### Protein array content

The protein array consists of a 96-dot format of proteins spotted onto nitrocellulose. There are 92 total *M. paratuberculosis *proteins recombinantly produced in *E. coli *with 89 of these using the pMAL-c2 expression vector (New England Biolabs), and 3 using the pET vector. All expression clones were sequenced to confirm that the cloned insert matched the native *M. paratuberculosis *gene and was in-frame with expression signals built into the expression vector. One spot on the array contains the MBP/LacZ protein used as a control to assess antibody reactivity to the MBP affinity tag. This control protein comprises the 42-kDa maltose binding protein along with the 8-kDa LacZ alpha peptide. Two spots contain a sonicated whole cell extract of *M. paratuberculosis *prepared as described previously [9] and one spot contains the PBS spotting buffer control only.

### Protein array production

PBS was used as the spotting buffer and diluent for all *M. paratuberculosis *proteins. All protein arrays were sequentially produced and no single array was used for more than one experiment. To print each array, the nitrocellulose membrane, pre-soaked in PBS, was placed over a rubber gasket such that it covered all wells of the Bio-dot 96-well manifold apparatus (BioRad, Hercules, CA). The top of the Bio-dot apparatus was then positioned over the membrane and fastened in each corner. Each well was pre-washed with 200 μl of PBS followed by a brief vacuum to pull the buffer through the membrane. Diluted working stocks of purified proteins were stored in deep 96-well plates with a 1-ml capacity per round bottom well (Nalgene Nunc International). Transfer of these proteins from the deep well plate to the assembled Bio-dot apparatus was performed using an 8-channel multi-pipette. Each well of the assembled 96-well dot blot apparatus was loaded with the appropriate protein at a final concentration of 3 μg/well as measured in a NanoDrop spectrophotometer (Thermo Fisher Scientific) at 280_nm_. The PBS-diluted samples were allowed to flow through the membrane by gravity flow. Each well was rinsed with 200 μl of PBS, 0.1% Tween-20 (TPBS). The vacuum was engaged to pull the rinse solution through and the nitrocellulose membrane was removed from the apparatus and placed in a Petri dish containing a blocking solution (TPBS-BSA; PBS with 2% bovine serum albumin, 0.1% Tween-20). After 1 hour in the blocking solution, the immunoblot assay was performed.

### Immunoblot analysis of the protein array

Serum from *M. paratuberculosis *infected calves were diluted in the blocking buffer and exposed to spotted arrays for two hours at room temperature on a rocker platform. All cattle sera were diluted 1:500. After three washes in TPBS, the nitrocellulose array was incubated for 1.5 h with Horse Radish Peroxidase-conjugated anti-bovine IgG antibody (Pierce) diluted at 1:20,000 in TPBS-BSA. This was followed by a final three washes in TPBS. Assay development was with SuperSignal detection reagents (Pierce) and Kodak BioMax MR film.

### Quantitative analysis of spot intensity

Spot intensity was measured using the Adobe Photoshop CS3 extended application. This version has the ability to record pixel gray values using the measurement scale of 1 pixel equals 1 pixel. Each spot was measured identically using a window area of 1804 pixels. Values were exported into a spreadsheet for further analysis. The background statistics were calculated by determining the mean and standard deviation of the 24 spots within each array that had the least signal intensity. Each intensity score was compared to the calculated background intensity. Values that were within two standard deviations of the background mean were set to zero and all other intensity values were subtracted from the background mean to give positive intensity values that were adjusted for the array background. To determine the reactivity distribution of all proteins in the complete set, the highest intensity score for each protein was examined as a way to determine if there was distinct stratification of maximal antibody response. Kernel Density Estimation performed on the log-transformed maximum intensity scores indicated there were three distinct normally distributed peaks. These distributions define the proteins belonging to the low, medium, or high reactivity groups presented in Figure 3.

## Authors' contributions

JPB conceived of the study, printed the protein arrays, participated in study design and coordination and drafted the manuscript. DOB provided bioinformatics support, statistical analysis and assisted with densitometric analyses of protein arrays. WRW and MVP conceived the experimental calf challenge model and performed the immunological characterizations. JRS carried out the immunoassays and determined immunological status for the subclinical cattle. MLP participated in the design of the study and performed bioinformatics analyses. All authors read and approved the final manuscript.
